# A novel *PAX5* rearrangement in *TCF3-PBX1* acute lymphoblastic leukemia: a case report

**DOI:** 10.1186/s12920-018-0444-9

**Published:** 2018-12-18

**Authors:** Thayana Conceição Barbosa, Bruno Almeida Lopes, Caroline Barbieri Blunck, Marcela Braga Mansur, Adriana Vanessa Santini Deyl, Mariana Emerenciano, Maria S. Pombo-de-Oliveira

**Affiliations:** 1 Division of Clinical Research, Research Center, Instituto Nacional de Câncer, Rio de Janeiro, RJ Brazil; 2 Pediatric Hematology-Oncology Program, Research Center, Instituto Nacional de Câncer, Rio de Janeiro, RJ Brazil; 3 Hospital de Clínicas de Porto Alegre, Porto Alegre, RS Brazil

**Keywords:** der(9)t(9;17)(p13;q11.2) translocation, *PAX5-SPECC1*, Near-triploidy karyotype, *TCF3-PBX1*

## Abstract

**Background:**

Chromosome translocations are a hallmark of B-cell precursor acute lymphoblastic leukemia (BCP-ALL). Additional genomic aberrations are also crucial in both BCP-ALL leukemogenesis and treatment management. Herein, we report the phenotypic and molecular cytogenetic characterization of an extremely rare case of BCP-ALL harboring two concomitant leukemia-associated chromosome translocations: t(1;19)(q23;q13.3) and t(9;17)(p13;q11.2). Of note, we described a new rearrangement between exon 6 of *PAX5* and a 17q11.2 region, where intron 3 of *SPECC1* is located. This rearrangement seems to disrupt *PAX5* similarly to a *PAX5* deletion. Furthermore, a distinct karyotype between diagnosis and relapse samples was observed, disclosing a complex clonal evolution during leukemia progression.

**Case presentation:**

A 16-year-old boy was admitted febrile with abdominal and joint pain. At clinical investigation, he presented with anemia, splenomegaly, low white blood cell count and 92% lymphoblast. He was diagnosed with pre-B ALL and treated according to high risk GBTLI-ALL2009. Twelve months after complete remission, he developed a relapse in consequence of a high central nervous system and bone marrow infiltration, and unfortunately died.

**Conclusions:**

To our knowledge, this is the first report of a rearrangement between *PAX5* and *SPECC1*. The presence of *TCF3-PBX1* and *PAX5-*rearrangement at diagnosis and relapse indicates that both might have participated in the malignant transformation disease maintenance and dismal outcome.

## Background

B-cell precursor acute lymphoblastic leukemia (BCP-ALL) is characterized by recurrent chromosomal alterations including translocations, which result in aberrant fusion genes. *TCF3-PBX1* is one of these fusions and is found in 3–6% of BCP-ALL patients; moreover, the acquisition of secondary genomic aberrations, such as loss of *CDKN2A/B*, *PAX5* or *RB1*, is common and crucial to leukemogenesis in those cases [1, 2].

*PAX5*, a master regulator of B-cell differentiation, is frequently disrupted by deletions, point mutations and amplifications in BCP-ALL. These disruptions might result in potentially oncogenic proteins [3, 4]. For example, *PAX5* amplifications (*PAX5*^AMP^) were identified in diagnostic-relapse matched samples of patients who lacked common cytogenetic abnormalities at relapse, indicating that *PAX5*^AMP^ may be an important driver of leukemogenesis [5]. Notably, *PAX5* can be labeled a “promiscuous” gene since over 16 partner *genes* have been identified in leukemia-associated rearrangements. These rearrangements result in fusion genes encoding chimeric proteins that modify PAX5 function and occur in 2–3% of pediatric BCP-ALL patients.

Here, we report an extremely rare case of BCP-ALL harboring two concomitant leukemia-associated alterations: *TCF3-PBX1* and *PAX5* rearrangement (*PAX5-*r). Additionally, to the best of our knowledge, this is the first report of *PAX5* disruption caused by a rearrangement with a 17q11.2 region, where *SPECC1* gene is located.

## Case presentation

### Clinical course

A 16-year-old boy was admitted to the Hospital das Clínicas de Porto Alegre, Porto Alegre, Brazil, febrile with abdominal and joint pain. At clinical investigation, he presented with anemia, splenomegaly and leukocytosis (white blood cells count 19.6 × 10^9^ /L) with 72% lymphoblast. Bone marrow (BM) aspiration disclosed lymphoblast cells infiltration (92%). The central nervous system (CNS) was not infiltrated by blast cells. The immunophenotyping was characterized by nTdT, cCD10, CD20, CD22, CD38 and CD45^(low)^ and cCD9, CD19, cCD79, and CD58^(interm)^-positive cells in 45% of blast cells. Myeloid and T-cell markers were negative. The patient was treated according to the GBTLI-ALL2009 at high-risk arm. He was a prednisone poor responder (at day 8; > 1000 circulating lymphoblasts), minimal residual disease at day 35 was negative, and he was considered in complete remission (CR). Twelve months after CR, he was hospitalized with a CNS infiltration and BM highly infiltrated with lymphoblasts. The laboratorial investigations demonstrated a similar immunophenotype profile and distinct karyotype. Despite undergoing the relapse treatment-rescue, the patient died due to complications from an opportunistic infection.

### Molecular analysis

The diagnosis and characterization of leukemia were established by morphology, immunophenotyping, and molecular-cytogenetic analysis according to the World Health Organization classification [6].

**Fig. 1:**
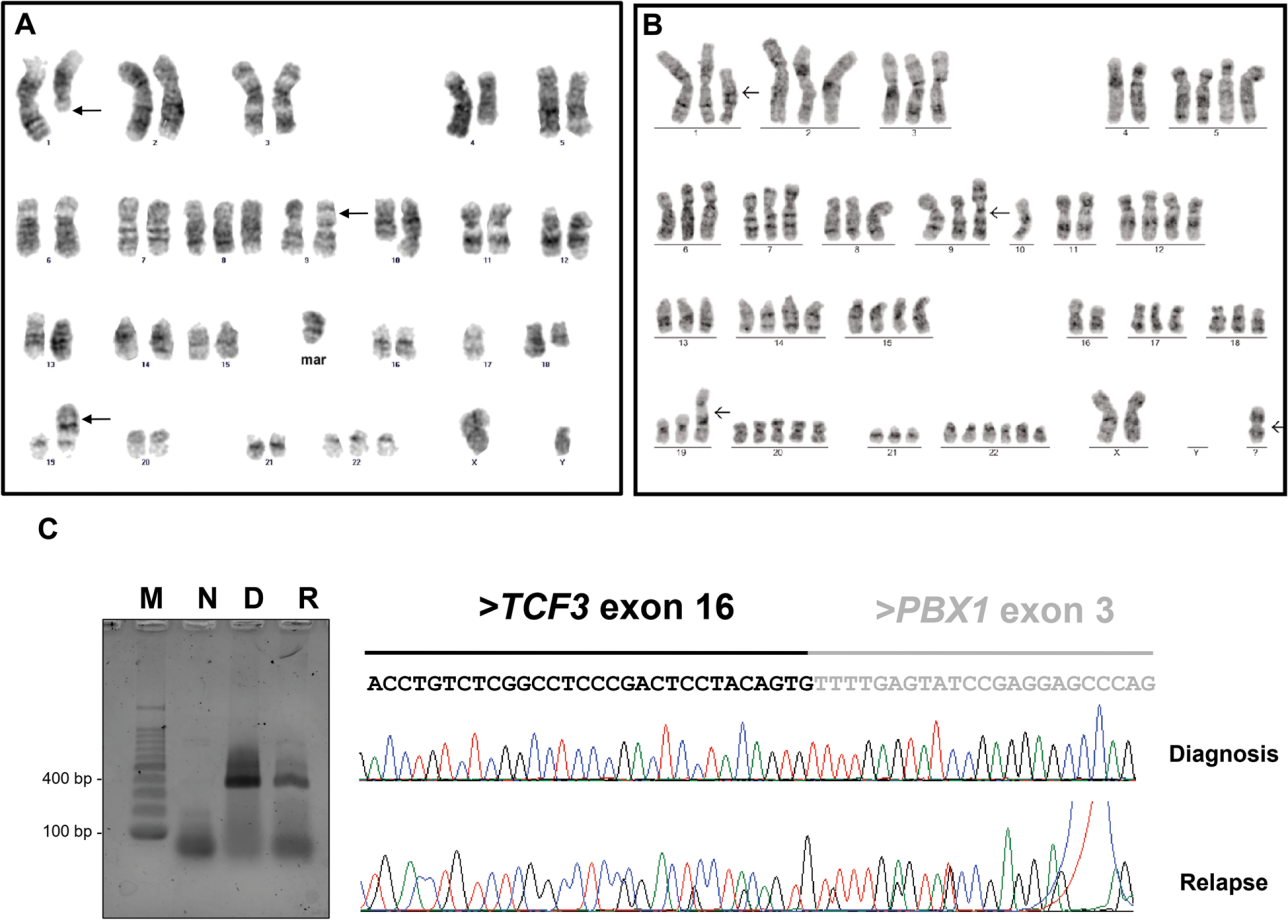
Karyotype and *TCF3-PBX1* confirmation. **a** Representative GTG-banded metaphase of the leukemic clone at diagnosis and **b** at relapse. **c** RT-PCR to *TCF3-PBX1* at diagnosis and relapse, respectively. M, marker (100pb); N, negative control; D, diagnosis sample; R, relapse sample. **d** Sequencing of truncated *TCF3-PBX1* at diagnosis and relapse

Cytogenetic analysis of leukemic BM was performed using GTG-banding standard procedures, and the karyotype was described according to the International System for Human Cytogenetic Nomenclature (ISCN) of 2013 [7, 8]. The karyotype of the diagnostic sample showed evidence of two concomitant chromosomal translocations (48,XY,t(1;19)(q23;q13.3),del(4)(q27q35),der(9)t(9;17)(p13;q11.2),del(10)(q24q26),del(18)(q21q3,+ 8,+ 22,+marc[20]) (Fig. 1a). In addition to the two rearrangements observed at diagnosis, the karyotype of the BM at relapse also showed near-triploidy: 73,XX,t(1;19)(q23;p13),+ 1,+ 2,+ 3,+ 5,+ 5,+ 6,+ 7,+ 8,i(9q),+ 9,10,+ 12,+ 12,+ 13,+ 14,+ 14,+ 15,+ 15,+ 17,+ 18,+ 19,+ 20,+ 20,+ 20,+ 21,+ 22,+ 22,+ 22,+ 22,+mar [2] (Fig. 1b). The presence of *TCF3-PBX1* was confirmed in both diagnostic and relapse samples by reverse transcription PCR (RT-PCR) followed by sequencing (Fig. 1c, d).

**Fig. 2:**
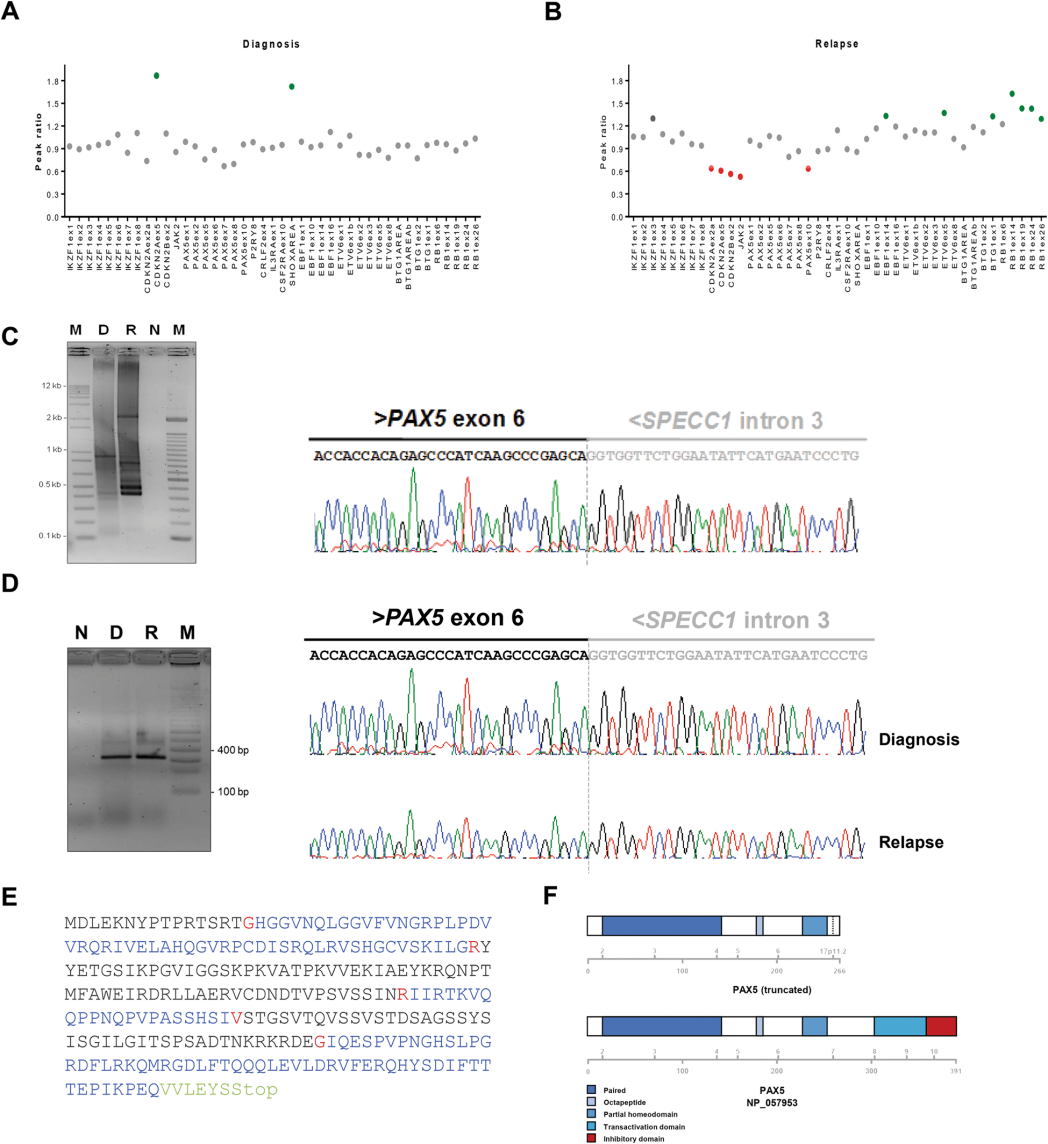
Characterization of additional abnormalities identified. **a, b** MLPA profile of matched sample of case described using the P335 ALL SALSA MLPA kit at diagnosis and relapse sample, respectively. **c** 3’RACE-PCR PAX5 and sequencing of truncated PAX5 after 3’RACE-PCR with diagnosis sample. M, marker (1 kb); N, negative control; D, diagnosis sample; R, relapse sample. **d** RT-PCR to *PAX5-SPECC1* and sequencing of *PAX5* transcript at diagnosis and relapse. **e** Expected protein sequence of the truncated PAX5 transcript derived from the *PAX5-SPECC1* head-to-head fusion. The translated protein refers to the PAX5–201 (NM_016734), showing its alternating exons (black and green) with splice acceptor amino acid residue (red), and amino acid residues coded from the fusion site on SPECC1 (green). **f** Schematic representation of the PAX5 wild-type protein and the fusion transcript detected

Additional copy number alterations (CNA) were identified by multiplex ligation-dependent probe amplification (MLPA) using the SALSA MLPA P335-A4/B1 and P202-B1 kits (MRC-Holland, Amsterdam, the Netherlands) as previously described [9]. MLPA data were analyzed using Coffalyser.Net. The relative copy number was obtained after the normalization of peaks against controls. Values between 0.70 and 1.3 were considered to be within the normal range. Values below 0.70 or above 1.3 indicated deletion or gain, respectively. Values below 0.25 indicated homozygous deletion. Submicroscopic deletions were only identified at relapse moment affecting *CDKN2A* (exons 2 and 5), *CDKN2B* (exon 2), *IKZF2* (exons 2 and 5) *JAK2* (exon 23), and miR31 (exon 1), as well as a partial gain in *RB1* (exons 14, 19, 24 and 26) (Fig. 2a, b).

To investigate the presence of a putative gene fusion derived from the PAX5-r we performed a 3’ Rapid amplification of cDNA ends (3’RACE)-PCR based on Scotto-Lavino’s protocol [10]. After cDNA synthesis, including an oligonucleotide (dT)-tailed primer (Q_T_), two rounds of PCR were performed to enrich the reaction for fragments containing the 3’end of *PAX5* transcripts. The first round of amplification used the forward primer PAX5.E4.F1 (5’-AACCAACCAGTCCCAGCTTC-3′), while the second was performed using the PAX5.E5.F1 primer (5’-TACTCCATCAGCGGCATCC-3′). The results showed a head-to-head fusion between exon 6 of *PAX5* and a 17q11.2 region, where intron 3 of *SPECC1* is located (Fig. 2c).

Subsequently, we used SuperScript™ III One-Step RT-PCR System (Invitrogen, Carlsbad, EUA) and sequencing on ABI®3500 Genetic Analyzer (Applied Biosystems, Foster City, EUA) to confirm that *PAX5*-r was present at both time points diagnosis and relapse (Fig. 2d). Furthermore, the predicted truncated PAX5 protein consisted of 266 amino acid residues, preserving the paired domain, the octapeptide motif and the homeodomain of PAX5, and a tail of 6 amino acids coded by the contiguous intron 3 sequence of *SPECC1*, which does not correspond to any predictive functional domain (Fig. 2e, f).

## Discussion and conclusions

In this case report, we describe the identification of a der(9)t(9;17)(p13;q11.2) translocation, resulting in *PAX5-*r, in a 16-year-old boy with *TCF3-PBX1* positive BCP-ALL. To date, this is the first report of a rearrangement between *PAX5*-exon 6 and *SPECC1*-intron 3. Sixteen *PAX5*-partner genes had been previously described, composing a heterogeneous group of genes encoding proteins that play distinct roles in signaling pathways, transcription regulation, chromatin remodeling and cell structure [3, 4]. However, in this case report, due to the absence of amino acid residues from *SPECC1* in the truncated protein formed, the effect of the new protein identified is likely to be similar to a *PAX5* recurrent deletion [11–13].

The *SPECC1* gene, also known as *HCMOGT*-*1*, has three different splicing variants and encodes the NSP5a3a protein, which belongs to the cytospin-A family. NSP5a3a is highly expressed in head and neck squamous cell carcinoma and in testis cell lines, but not in other normal cells [14]. Furthermore, NSP5a3a interacts with the nucleolar phosphoprotein B23, which plays multiple roles in cell growth and proliferation [15]. To the best of our knowledge, the only report of *SPECC1* altered in the leukemic context was a case of juvenile myelomonocytic leukemia with *PDGFRB-SPECC1* [16].

With regard to other CNAs and in concordance with previous findings in *PAX5-*rearranged leukemias, few deletions and/or gains are observed [3, 4]. Here, we detected a heterozygous deletions affecting *CDKN2A/B*, *JAK2* and miR31, confirming the presence of an unbalanced t(9;17) with 9p13.2-pter deletion. *CDKN2A/B* deletions are common in ALL and frequently observed in concomitance with other *PAX5* gene fusions, such as *PAX5-C20orf112* and *PAX5-ETV6* [4]*.* Regarding *TCF3-PBX1* patients, the additional CNAs observed in this case report are in fact the most frequent alterations found in this cytogenetic-subtype. Furthermore, they are known to have a cumulative effect on the leukemic development [17]. Our analysis of CNAs at diagnosis and relapse revealed a high degree of clonal heterogeneity and a complex evolution between diagnosis and relapse samples, suggesting a clonal selection pattern during leukemia progression, which could have implications for the treatment efficiency. Moreover, based on the FMC characterization, we assume that the major clone harboring a *TCF3-PBX1* fusion. Although there is no description of BCP-ALL subsets associated with *PAX5*- r, the presence of a second clone is not discharged and would be explored.

In conclusion, although the occurrence of near-triploidy has been found in BCP-ALL *TCF3-PBX1* patients, the concomitant presence of *PAX5*-r should be revisited. The translocation *PAX5-SPECC1* is a new report that requires further investigations. We also showed that both *TCF3-PBX1* and *PAX5-*r were present at diagnosis and relapse, indicating that both might have participated in the malignant transformation disease maintenance and dismal outcome.

## Abbreviations

3’RACE: 3’ Rapid amplification of cDNA ends
BCP-ALL: B-cell precursor acute lymphoblastic leukemia
BM: Bone marrow
CNA: Copy number alterations
CNS: Central nervous system
CR: Complete remission
ISCN: International System for Human Cytogenetic Nomenclature
MLPA: Multiplex ligation-dependent probe amplification
*PAX5*^AMP^: *PAX5* amplifications
RT-PCR: Reverse transcription PCR

## References

[1] Barbosa TC, Mansur MB, Blunck CB, Emerenciano M, Pombo-de-Oliveira MS (2017). Characterization of RB1 in pediatric TCF3-PBX1+ acute lymphoblastic leukemia. Blood.

[2] Duque-Afonso J, Feng J, Scherer F, Lin CH, Wong SH, Wang Z, Iwasaki M, Cleary ML (2015). Comparative genomics reveals multistep pathogenesis of E2A-PBX1 acute lymphoblastic leukemia. J Clin Invest.

[3] Nebral K, Denk D, Attarbaschi A, König M, Mann G, Haas OA, Strehl S (2009). Incidence and diversity of PAX5 fusion genes in childhood acute lymphoblastic leukemia. Leukemia.

[4] Coyaud E, Struski S, Prade N, Familiades J, Eichner R, Quelen C (2010). Wide diversity of PAX5 alterations in B-ALL: a Groupe francophone de Cytogenetique Hematologique study. Blood.

[5] Schwab C, Nebral K, Chilton L, Leschi C, Waanders E, Boer JM (2017). Intragenic amplification of PAX5: a novel subgroup in B-cell precursor acute lymphoblastic leukemia?. Blood Adv.

[6] Arber DA, Orazi A, Hasserjian R, Thiele J, Borowitz MJ, Le Beau MM (2016). The 2016 revision to the World Health Organization classification of myeloid neoplasms and acute leukemia. Blood.

[7] Yunis JJ (1982). Comparative analysis of high-resolution chromosome techniques for leukemic bone marrows. Cancer Genet Cytogenet.

[8] Shaffer LG, McGowan-Jordan J, Schmid M (2013). An international system for human cytogenetic nomenclature. Recommendations of the international standing committee on human cytogenetic nomenclature.

[9] Barbosa TC, Terra-Granado E, Quezado Magalhães IM, Neves GR, Gadelha A, GuedesFilho GE (2015). Frequency of copy number abnormalities in common genes associated with B-cell precursor acute lymphoblastic leukemia cytogenetic subtypes in Brazilian children. Cancer Genet.

[10] Scotto-Lavino E, Du G, Frohman MA (2006). 3’ end cDNA amplification using classic RACE. Nat Protoc.

[11] Artimo P, Jonnalagedda M, Arnold K, Baratin D, Csardi G, de Castro E, Duvaud S (2012). ExPASy: SIB bioinformatics resource portal. Nucleic Acids Res.

[12] Fortschegger K, Anderl S, Denk D, Strehl S (2014). Functional heterogeneity of PAX5 chimeras reveals insight for leukemia development. Mol Cancer Res.

[13] Kawamata N, Pennella MA, Woo JL, Berk AJ, Koeffler HP (2012). Dominant-negative mechanism of leukemogenic PAX5 fusions. Oncogene.

[14] Sang N, Fath DM, Giordano A (2004). A gene highly expressed in tumor cells encodes novel structure proteins. Oncogene.

[15] D'Agostino L, Giordano A (2008). Possible functional role of NSPs in cancer. Cell Cycle.

[16] Morerio C, Acquila M, Rosanda C, Rapella A, Dufour C, Locatelli F (2004). HCMOGT-1 is a novel fusion partner to PDGFRB in juvenile myelomonocytic leukemia with t(5;17)(q33;p11.2). Cancer Res.

[17] Andersen MK, Autio K, Barbany G, Borgström G, Cavelier L, Golovleva I (2011). Paediatric B-cell precursor acute lymphoblastic leukaemia with t(1;19)(q23;p13): clinical and cytogenetic characteristics of 47 cases from the Nordic countries treated according to NOPHO protocols. Br J Haematol.

